# A Deep Learning-Assisted Multi-Relay DCSK Communication System

**DOI:** 10.3390/s26082420

**Published:** 2026-04-15

**Authors:** Tingting Huang, Shengmin Hong, Jundong Chen, Liangyi Kang

**Affiliations:** School of Engineering, Huaqiao University, Quanzhou 362021, China; tthuang@hqu.edu.cn (T.H.); jdchan0815@163.com (J.C.); 24014084010@stu.hqu.edu.cn (L.K.)

**Keywords:** chaotic communication, channel quality, DCSK, deep learning, V2V

## Abstract

This paper proposes a novel multi-relay deep learning-assisted differential chaos shift keying (MR-DL-DCSK) communication system to enhance the capabilities of existing chaos-based cooperative communication systems. Channel quality significantly affects transmission reliability. However, existing channel quality evaluation methods require channel state information (CSI). To address this limitation, a deep neural network (DNN) classifier is employed at the receiver in this paper to perform joint channel quality assessment and symbol demodulation. We propose a channel quality-aware relay coordination strategy: at the relay stage, all relays assess their channel qualities using the DNN-output probability distribution, and relays with lower channel quality align their decoded bits with the bits from the relay with the highest channel quality before forwarding; at the destination stage, the destination selects the signal with the highest channel quality probability for final demodulation. This joint detection approach enables reliable demodulation without requiring explicit CSI, while the channel quality-aware relay coordination mechanism ensures that signals from the most reliable links are prioritized for final decision. Comprehensive simulation results demonstrate that the proposed multi-relay DL-DCSK system achieves superior bit error rate performance. Furthermore, the system exhibits excellent generalization capability when tested on vehicle-to-vehicle (V2V) communication channels modeled by the double-generalized gamma distribution, validating its practical applicability in diverse wireless environments.

## 1. Introduction

As an important part of wireless communication, cooperative communication has achieved rapid development in the past decades [1,2]. Among the enabling technologies in this domain, relay-assisted transmission has proven particularly effective at enhancing both link reliability and network coverage [3,4,5].

Owing to their inherent characteristics of non-periodicity, noise-like behavior, and high sensitivity to initial conditions, chaotic signals have recently gained widespread attention in communication systems. These properties make chaotic signals well-suited for applications that demand physical-layer security and resilience against interference [6,7]. Consequently, chaotic communication techniques have been deployed across a broad range of application domains, including high-speed railway systems [8], underwater acoustic channels [9,10], optical links [11], and Internet-of-Things (IoT) networks [12]. In particular, differential chaos shift keying (DCSK) [13], which eliminates the need for channel state information (CSI) at the receiver, has been extensively studied in cooperative communication systems. More recently, the growing maturity of deep learning (DL) has opened new avenues for performance enhancement, with researchers increasingly integrating DL-based techniques into both chaotic and cooperative communication systems.

### 1.1. Related Works

Building on these advances, prior studies have demonstrated that integrating DCSK/FM-DCSK modulation with cooperative relaying strategies yields substantial practical benefits for wireless communication networks [14]. To improve spectral efficiency, a partial-sequence cooperative communication (PS-CC) scheme for DCSK-based cooperative systems was proposed in [15], achieving higher data rates while reducing computational burden at the relay nodes. Addressing bidirectional communication, a two-relay two-way DCSK system was presented in [16], in which all relay nodes adopt the decode-and-forward (DF) protocol to process signals received from both user terminals. From the perspective of energy harvesting, a buffer-aided relay system combining DCSK modulation with simultaneous wireless information and power transfer (SWIPT) was investigated in [17], yielding improved bit error rate (BER) performance and reduced end-to-end latency. To further push throughput boundaries, a code-index-modulation-assisted short-reference DCSK cooperative communication (CIM-SR-DCSK-CC) system was proposed in [18], demonstrating significant throughput gains without any degradation in BER performance.

The advancement of deep learning (DL) has significantly propelled progress across multiple communication research frontiers [19]. By leveraging powerful feature extraction and nonlinear modeling capabilities, DL offers fresh perspectives on problems that have long resisted conventional analytical approaches [20,21]. Building upon this potential, DL-driven relay selection and resource optimization have emerged as particularly impactful directions for advancing cooperative communication networks. In [22], a deep neural network (DNN) was employed to jointly optimize relay selection and power allocation, enabling the simultaneous handling of both tasks across the source and individual relays—a level of complexity that conventional algorithms are unable to achieve. Complementarily, a DNN-based relay selection scheme proposed in [23] addresses wireless-powered cognitive IoT networks, delivering accurate throughput prediction and relay selection decisions under reduced channel feedback overhead and tight latency constraints. Extending these ideas to wireless sensor networks (WSNs), the study in [24] introduced a deep reinforcement learning (DRL)-based relay selection scheme, achieving lower outage probability, higher system capacity, and reduced energy consumption with faster convergence compared to conventional methods. In a different propagation environment, [25] developed a DRL-based adaptive relay selection and power allocation scheme for cooperative free-space optical (FSO) communication systems, maintaining robust system performance across a wide range of atmospheric attenuation and turbulence conditions.

Beyond relay selection and resource optimization, DL has also been successfully integrated into transceiver design, with chaotic communication systems emerging as a particularly active application domain. Early efforts in this direction introduced a DL-aided OFDM-DCSK transceiver architecture capable of performing intelligent signal demodulation at the receiver [26,27]. Building on this foundation, a neural network-aided detection technique for index modulation DCSK (NN-IM-DCSK) was developed in [28] to further enhance transmission reliability. Targeting more complex modulation schemes, the work in [29] proposed a multilevel code shifted *M*-ary differential chaos shift keying (MCS-MDCSK) system, in which a DNN-based detector jointly performs demodulation and despreading at the receiver. More recently, a power spectral density-based deep learning chaos shift keying (PSD-DLCSK) scheme was introduced in [30], which exploits the inherent spectral characteristics of chaotic signals to achieve superior BER performance. Collectively, these studies demonstrate that DL-based transceivers, by harnessing the feature extraction power of neural networks, can substantially improve the reliability of chaotic communication systems—thereby motivating their further integration into multi-relay DCSK frameworks.

### 1.2. Motivation and Contributions

Although DCSK-based cooperative relay systems and DL-assisted communication designs have demonstrated notable improvements in transmission reliability, channel quality remains an important factor in further influencing transmission reliability and overall system performance [31]. The practical significance of channel quality has been evidenced in several studies. In industrial D2D-enabled small cell networks, the introduction of a channel quality factor (CQF) has been shown to ensure uninterrupted low-latency communication while enabling dynamic parameter adjustment to improve data rate and reliability [32]. Similarly, a composite channel quality metric derived from physical layer (PHY) measurements was proposed in [33], exhibiting a strong linear correlation with application-layer quality of service (QoS) indicators such as packet error rate (PER). These findings collectively underscore that reliable channel quality assessment is indispensable for optimizing system performance. Motivated by this, DL has been explored as a means of achieving accurate and efficient channel quality evaluation [34].

Although considerable research effort has been devoted to enhancing cooperative communication systems through DL, its application to chaotic cooperative communication—multi-relay DCSK communication systems in particular—remains largely unexplored. Motivated by this gap, this paper proposes a multi-relay DL-assisted DCSK (MR-DL-DCSK) communication system, in which a deep neural network (DNN) is embedded at the receiver as an intelligent classifier to jointly perform channel quality assessment and symbol demodulation. By leveraging the pattern recognition capability of DL, the proposed system implicitly estimates instantaneous channel conditions during symbol demodulation—without requiring explicit channel state information—thereby enabling adaptive relay coordination and robust data recovery in dynamic wireless environments.

To further exploit the channel quality information provided by the DNN, we propose a novel channel quality-aware relay coordination strategy, distinct from traditional relay forwarding protocols [35] and conventional diversity combining techniques [16]. At the relay stage, each relay evaluates its channel quality using the class probability distribution output by the DNN. Relays experiencing inferior channel conditions then align their decoded bits with those of the relay reporting the highest channel quality prior to forwarding. At the destination stage, the receiver selects the signal corresponding to the highest channel quality probability for final demodulation. The bit alignment process ensures the consistency of information forwarded across all relay nodes, effectively suppressing error propagation introduced by low-quality links. Meanwhile, the data-driven signal selection at the destination further enhances end-to-end transmission reliability without relying on explicit channel state information.

The main contributions of this paper are summarized as follows:

- We propose a novel multi-relay DL-DCSK system that integrates a DNN-based intelligent classifier for joint channel quality evaluation and symbol detection. During offline training, channel quality labels are derived from BER statistics, with low-BER conditions categorized as high quality and high-BER conditions as low quality. The resulting probability distribution output by the DNN then serves as the basis for quality-aware relay coordination during online deployment;
- We propose a novel channel quality-aware relay coordination strategy that departs from both traditional relay forwarding protocols and conventional diversity combining techniques. Specifically, the strategy employs a relay bit alignment mechanism to ensure that all relay nodes forward consistent information anchored to the most reliable relay, while the destination performs signal selection guided by the DNN-output channel quality probability distribution. Together, these two mechanisms suppress error propagation from low-quality links and enable data-driven signal selection, collectively improving end-to-end transmission reliability without requiring explicit channel state information;
- We conduct comprehensive performance evaluations over multipath Rayleigh fading channels and realistic vehicle-to-vehicle (V2V) channels modeled by the double-Gamma distribution. Simulation results demonstrate that the proposed multi-relay DL-DCSK system consistently outperforms the conventional TWRNC-DCSK benchmark and the FC-DNN-based detection method across all evaluated scenarios. Furthermore, ablation experiments across different relay coordination strategies validate the effectiveness of the proposed channel quality-aware coordination mechanism. The system maintains robust BER performance across varying spreading factors, relay counts, and power-delay profiles, with performance improving monotonically as the number of relays increases. Moreover, simulations under high-mobility V2V conditions at a relative vehicle speed of 80 km/h confirm that the proposed system maintains robust performance in the presence of Doppler-induced channel variations. Finally, practical detection latency measurements further confirm that the computational overhead of the proposed DNN-based receiver is manageable and can be substantially reduced through modern inference optimization techniques.

The remainder of this paper is organized as follows. Section 2 describes the system model of the proposed multi-relay DL-DCSK system, including the transmitter structure, relay protocol, and DNN-based receiver design. Section 3 details the architecture of the DNN classifier, the training process, hyperparameter selection, and complexity analysis with practical detection latency measurements. Section 4 presents the simulation results and discussions, analyzing the BER performance under different system configurations, channel conditions, relay coordination strategies, and high-mobility V2V scenarios with Doppler effects. Finally, Section 5 concludes the paper and suggests future research directions.

## 2. System Model

### 2.1. DL-DCSK System

**Figure 1:**
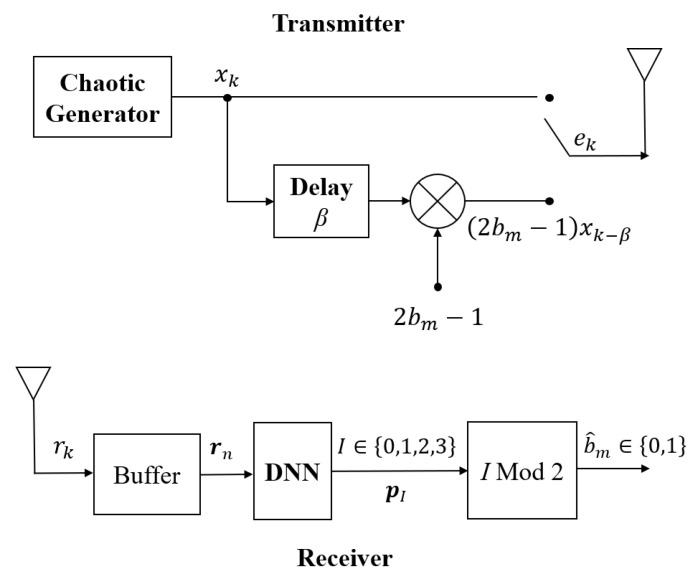
Block diagram of the DL-DCSK transceiver.

The transceiver structure of the proposed DL-DCSK system is illustrated in Figure 1. At the transmitter, each information bit $b_{m}\in \left\{0,1\right\}$ is represented by two chaotic sequences of equal length β, where *m* denotes the bit index and β is the spreading factor. Specifically, the transmission of each bit is divided into two half-bit durations. In the first half-bit duration, a chaotic sequence is transmitted as a reference signal; in the second half, the same sequence is forwarded if the bit is “1”, whereas its inverted version is sent if the bit is “0”. In this paper, the logistic map is adopted as the chaotic generator owing to its simplicity [16] and its desirable property of eliminating autocorrelation [36]: $x_{k+1}=1-2x_{k}^{2}$, where $x_{k}$(k=1,2,…) denotes the *k*th chip of the chaotic sequence. The *k*th chip of the transmitted signal corresponding to the *m*th bit is expressed as

(1) $$e_{k}=\left\{\begin{array}{cc} x_{k}, & k=2(m-1)\beta +1,\ldots ,2(m-1)\beta +\beta \\ \left(2b_{m}-1\right)x_{k-\beta }, & k=2(m-1)\beta +1,\ldots ,2m\beta . \end{array}\right.$$

At the receiver, a DNN-based intelligent demodulator is proposed to replace the conventional correlation-based demodulation approach. After propagating through the multipath fading channel, the transmitted signal is subject to both channel noise and fading distortion. The received signal can be expressed as

(2) $$r_{k}=\sum_{l=1}^{L}\alpha_{l}e_{k-\tau_{l}}+z_{k},$$

where $\alpha_{l}$ and $\tau_{l}$ denote the fading coefficient and the delay of the *l*th path, and $z_{k}$ is the AWGN with zero mean and variance $N_{0}/2$.

The received chips are buffered across all 2β chip slots and assembled into the information-bearing sequence $r_{m}=\left[r_{0},r_{1},\ldots ,r_{k},\ldots ,r_{2\beta -1}\right]$. The sequence $r_{m}$ is then fed into the pre-trained DNN, which performs feature extraction and classification to produce two outputs: the predicted label I∈{0,1,2,3}, whose definition will be elaborated in Section 2.2.2, and the class probability distribution $p_{I}=\left[p_{I=0},p_{I=1},p_{I=2},p_{I=3}\right]$. Finally, the demodulated bit ${\hat{b}}_{m}$ is recovered by applying a modulo-2 operation to the predicted label *I*.

### 2.2. Multi-Relay DL-DCSK System

#### 2.2.1. Working Principle of Multi-Relay DL-DCSK System

**Figure 2:**
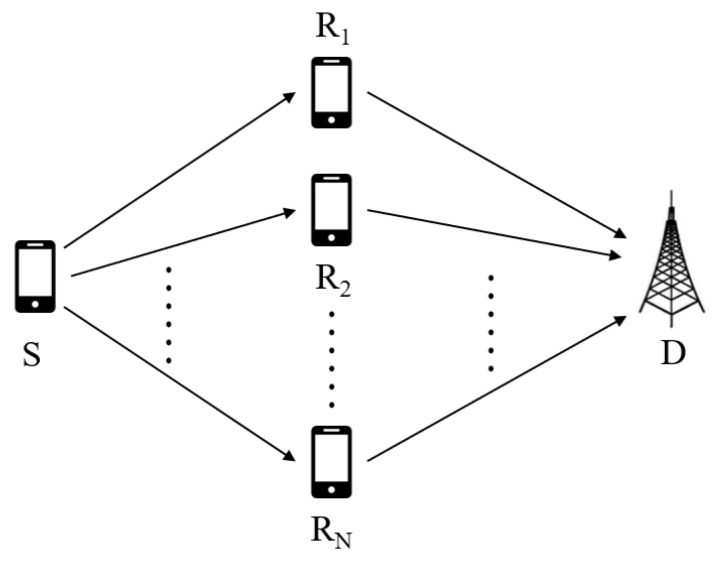
System model of the multi-relay DL-DCSK system.

The proposed multi-relay DL-DCSK system, as illustrated in Figure 2, consists of a source *S*, a destination *D*, and *N* relay nodes $\left\{R_{1},R_{2},\ldots ,R_{N}\right\}$. It is assumed that no direct link exists between the source and the destination.

Similar to time division multiple access (TDMA), to avoid interference, the multi-relay DL-DCSK system operates in two phases. In the first phase, the source *S* broadcasts DCSK-modulated signals to all *N* relay nodes. Upon reception, each relay employs the DNN-based receiver (Figure 1) to jointly demodulate the received signal and assess the quality of the source-to-relay channel. Prior to forwarding, all relays exchange their channel quality indicators and decoded bits via a lightweight inter-relay signaling mechanism. Relays with inferior channel quality then align their decoded bits with those of the relay reporting the highest channel quality, after which all relays forward their aligned bits to the destination. In the second phase, the destination *D* receives the forwarded signals from all relay nodes in successive time slots, and applies the same DNN-based receiver to assess the relay-to-destination channel quality for each received signal. The destination then selects the signal with the highest channel quality probability and applies a modulo-2 operation to its corresponding predicted label *I* to recover the final demodulated bit. The above two-phase procedure—encompassing relay bit alignment at the relay stage and quality-based signal selection at the destination—is collectively referred to as the channel quality-aware relay coordination strategy.

The detailed signal processing procedures, channel quality estimation methodology, inter-relay signaling mechanism, and associated signaling overhead are elaborated in the following two subsections.

#### 2.2.2. Signal Processing and Channel Quality Estimation

As established in [31], channel quality varies across different link conditions and can be quantified by the bit error rate (BER). To improve transmission reliability, a DNN is pre-trained to jointly perform channel quality assessment and transmitted bit estimation. In the offline training phase, channel quality is evaluated by computing the BER of the channel through which the received signal $r_{m}$ has propagated, and channels are classified into two categories: high quality and low quality. The resulting class labels, together with the corresponding transmitted bits, are used as training targets for the DNN. The specific label assignments are summarized in Table 1. During online deployment, the pre-trained DNN simultaneously estimates the transmitted bit and assesses the channel quality of each received signal. The DNN training process and the channel classification methodology are elaborated in Section 3.3.1, while the signal processing procedures for each transmission phase are described as follows.

**Phase 1: Source to Relays**

The source *S* broadcasts DCSK-modulated signals to all *N* relay nodes. The received signal at the *n*th relay follows the channel model in (2) with subscript $SR_{n}$, i.e.,

(3) $$r_{SR_{n},k}=\sum_{l=1}^{L_{SR_{n}}}\alpha_{SR_{n},l}e_{SR_{n},k-\tau_{SR_{n},l}}+z_{SR_{n},k},$$

where $\alpha_{SR_{n},l}$ and $\tau_{SR_{n},l}$ (n∈{1,2,…,N}) denote the fading coefficient and the delay of the *l*th path, respectively, and $z_{SR_{n},k}$ denotes the additive white Gaussian noise (AWGN) with zero mean and variance $N_{0}/2$ for the $S\to R_{n}$ link.

Following the signal processing procedure described in Section 2.1, each relay $R_{n}$ assembles the information-bearing sequence $r_{SR_{n}}=\left[r_{SR_{n},0},r_{SR_{n},1},\ldots ,r_{SR_{n},k},\ldots ,r_{SR_{n},2\beta -1}\right]$ and feeds it to the DNN-based receiver (Figure 1). The DNN outputs the predicted label $I^{SR_{n}}$ and the corresponding probability distribution $p_{I}^{SR_{n}}$ = $\left[p_{I=0}^{SR_{n}},p_{I=1}^{SR_{n}},p_{I=2}^{SR_{n}},p_{I=3}^{SR_{n}}\right]$.

To assess the channel quality, each relay $R_{n}$ calculates the high-quality channel probability as

(4) $$p_{high}^{SR_{n}}=p_{I=0}^{SR_{n}}+p_{I=1}^{SR_{n}}.$$

All relays compare their channel quality probabilities $\left\{p_{high}^{SR_{1}},p_{high}^{SR_{2}},\ldots ,p_{high}^{SR_{N}}\right\}$ to identify the relay with the highest channel quality. Let $R_{j}$ denote the relay with the maximum $p_{high}^{SR_{n}}$:

(5) $$j=\arg\max_{n\in \{1,2,\ldots ,N\}}p_{high}^{SR_{n}}.$$

Relay $R_{j}$ demodulates its bit as

(6) $${\hat{b}}_{m}^{SR_{j}}=I^{SR_{j}}\;\mathrm{mod}\;2.$$

The relays with lower channel quality ($p_{high}^{SR_{n}}<p_{high}^{SR_{j}}$) adjust their decoded bits to align with ${\hat{b}}_{m}^{SR_{j}}$. Consequently, all relays forward the same bit ${\hat{b}}_{m}^{R}={\hat{b}}_{m}^{SR_{j}}$ to the destination using DCSK modulation in different time slots.

**Phase 2: Relays to Destination**

In the second phase, the destination *D* receives forwarded signals from all relays in successive time slots. The received signal from the *n*th relay at the destination follows

(7) $$r_{R_{n}D,k}=\sum_{l=1}^{L_{R_{n}D}}\alpha_{R_{n}D,l}e_{R_{n},k-\tau_{R_{n}D,l}}+z_{R_{n}D,k},$$

where $\alpha_{R_{n}D,l}$ and $\tau_{R_{n}D,l}$ (n∈{1,2,…,N}) denote the fading coefficient and the delay of the *l*th path, respectively, and $z_{R_{n}D,k}$ denotes the additive white Gaussian noise (AWGN) with zero mean and variance $N_{0}/2$ for the $R_{n}\to D$ link.

The destination applies the same DNN-based receiver shown in Figure 1 to process each received signal $r_{R_{n}D}=\left[r_{R_{n}D,0},r_{R_{n}D,1},\ldots ,r_{R_{n}D,k},\ldots ,r_{R_{n}D,2\beta -1}\right]$, obtaining the predicted label $I^{R_{n}D}$ and probability distribution $p_{I}^{R_{n}D}=\left[p_{I=0}^{R_{n}D},p_{I=1}^{R_{n}D},p_{I=2}^{R_{n}D},p_{I=3}^{R_{n}D}\right]$ for each relay-to-destination link.

The destination evaluates the channel quality for each received signal by computing

(8) $$p_{high}^{R_{n}D}=p_{I=0}^{R_{n}D}+p_{I=1}^{R_{n}D}.$$

The destination then selects the received signal with the highest channel quality probability:

(9) $$j^{*}=\arg\max_{n\in \{1,2,\ldots ,N\}}p_{high}^{R_{n}D}.$$

Finally, the destination demodulates the final bit from the selected signal $r^{R_{j^{*}}D}$ as

(10) $${\hat{b}}_{m}=I^{R_{j^{*}}D}\;\mathrm{mod}\;2,$$

which serves as the demodulated bit of the multi-relay DL-DCSK system.

#### 2.2.3. Inter-Relay Signaling Mechanism and Overhead

The inter-relay signaling mechanism operates as follows. Upon completing DNN-based demodulation, each relay $R_{n}$ obtains its high-quality channel probability $p_{\mathrm{high}}^{SR_{n}}$ and decoded bit ${\hat{b}}_{m}^{SR_{n}}$, and broadcasts these two quantities to all other relays over a low-rate control channel. Since the system already operates under a TDMA framework, a dedicated coordination slot is inserted between the two transmission phases to facilitate this exchange. During this slot, each relay broadcasts one probability value and one binary bit. Each relay then independently identifies the relay with the highest $p_{\mathrm{high}}^{SR_{n}}$ and aligns its decoded bit accordingly, without requiring a centralized controller.

The signaling overhead of this coordination process is minimal: for an *N*-relay system, the total exchange per symbol period comprises only *N* probability values and *N* binary bits, which is negligible relative to the 2β-chip data payload of each DCSK symbol. Furthermore, synchronization among relay nodes is inherently ensured by the TDMA structure, as each relay operates within its pre-assigned time slot.

## 3. DNN-Based Intelligent Classifier

This section presents the architecture of the proposed DNN-based intelligent classifier, with a detailed description of each layer’s function. The hyperparameter selection criteria and computational complexity analysis are also discussed.

### 3.1. DNN Architecture

**Figure 3:**
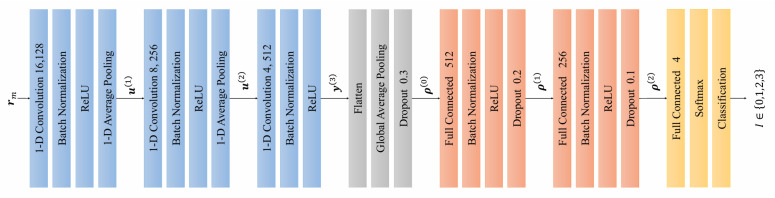
The architecture of DNN-based intelligent classifier.

Figure 3 illustrates the architecture of the proposed DNN-based intelligent classifier, which comprises an input layer, three one-dimensional convolutional layers (1D-CLs), five batch normalization (BN) layers, two one-dimensional average pooling layers (1D-APLs), a flatten layer, a global average pooling (GAP) layer, three dropout layers, three fully connected (FC) layers, a softmax layer, and an output classification layer. As described in Section 2, the information-bearing sequence $r_{m}$ is fed into the DNN to simultaneously perform channel quality assessment and symbol demodulation.

At the input layer, the received signal $r_{m}$ is first processed by the first convolutional layer to capture local features and short-term patterns of the signal. This convolutional layer employs 128 filters with a kernel size of 16, enabling the effective extraction of preliminary feature representations from the input. The subsequent BN layer normalizes the convolutional output, thereby accelerating training convergence and stabilizing gradient propagation. A ReLU activation function is then applied to introduce nonlinearity, enhancing the representational capacity of the network. The subsequent 1D-APL performs downsampling to reduce feature dimensionality, preserving salient information while lowering the computational overhead.

The feature maps produced by the first convolutional module are subsequently fed into the second 1D-CL for deeper feature extraction. This layer employs 256 filters with a kernel size of 8, enabling the extraction of more abstract signal representations and higher-level channel quality indicators. As in the first module, this convolutional layer is followed by a BN layer, ReLU activation, and a 1D-APL to achieve further feature refinement and dimensionality reduction.

The third 1D-CL employs 512 filters with the smallest kernel size of 4 to extract the highest-level semantic feature representations. Through the cascaded processing of three convolutional modules, the network progressively extracts multilevel feature representations, spanning from low-level waveform characteristics to high-level semantic abstractions of the raw DCSK signal. Furthermore, BN layers are inserted after each convolutional module to mitigate overfitting and improve generalization performance.

Following the convolutional modules, the flatten layer reshapes the multi-dimensional feature maps into a one-dimensional feature vector, while the GAP layer computes a global average over each feature map, substantially reducing the parameter count and mitigating overfitting. The resulting feature vector is then passed through three successive FC modules. The first FC layer contains 512 neurons and is coupled with a BN layer and a dropout layer with a dropout rate of 30%, enabling high-dimensional feature mapping. The second FC layer contains 256 neurons with a dropout rate of 20%, progressively compressing the feature space. The third FC layer applies a dropout rate of 10% and produces 4 output neurons, each corresponding to one of the four classification categories.

Finally, the softmax layer converts the output of the third FC layer into a class probability distribution $p_{I}$, where each element represents the likelihood of the input belonging to one of the four classes. These four classes correspond to the four combinations of transmitted bit value {0,1} and channel quality condition {high,low} as detailed in Table 1. The output classification layer then assigns the input to the class with the highest probability, yielding a joint prediction of channel quality and transmitted bit.

### 3.2. DNN Layers

#### 3.2.1. Convolutional Modules

**Figure 4:**
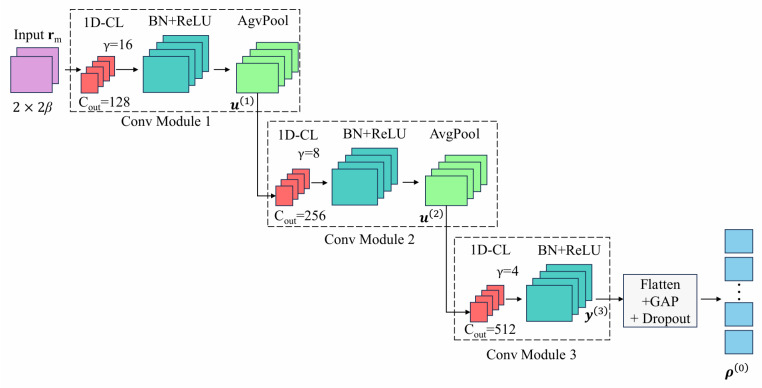
Working process of Conv Modules and GAP Module.

The DNN-based classifier employs three cascaded convolutional modules for hierarchical feature extraction as illustrated in Figure 4. Each module comprises a 1D-CL, a BN layer, and a ReLU activation function. The first and second modules additionally incorporate a 1D-APL for downsampling and dimensionality reduction.

**1D Convolutional Layer (1D-CL):** The 1D-CL performs convolution operations on the input feature maps to extract local temporal features. For the *q*-th convolutional module (q=1,2,3), let $u^{\left(q-1\right)}$ denote the input feature map, where $u^{\left(0\right)}=r_{m}$ represents the received signal. The convolution operation can be expressed as

(11) $$v_{d,t}^{\left(q\right)}=\sum_{c=0}^{C_{in}^{\left(q\right)}-1}\sum_{s=0}^{\gamma^{\left(q\right)}-1}W_{d,c,s}^{\left(q\right)}\cdot u_{c,t+s}^{\left(q-1\right)}+\Phi_{d}^{\left(q\right)}$$

where $v_{d,t}^{\left(q\right)}$ denotes the output of the *d*-th convolutional kernel at time position *t* in the *q*-th module, $W_{d,c,s}^{\left(q\right)}$ is the weight connecting the *c*-th input channel at position *s* to the *d*-th output channel, $\Phi_{d}^{\left(q\right)}$ is the bias term for the *d*-th kernel, $\gamma^{\left(q\right)}$ is the kernel size, and $C_{in}^{\left(q\right)}$ is the number of input channels.

**Batch Normalization (BN):** After convolution, the BN layer normalizes the feature maps to accelerate training and improve generalization:

(12) $${\hat{v}}_{d,t}^{\left(q\right)}=\frac{v_{d,t}^{\left(q\right)}-E\left[v_{d}^{\left(q\right)}\right]}{\sqrt{\mathrm{Var}[v_{d}^{\left(q\right)}]+\varepsilon }}$$

(13) $${\tilde{v}}_{d,t}^{\left(q\right)}=\lambda_{d}^{\left(q\right)}\cdot {\hat{v}}_{d,t}^{\left(q\right)}+\psi_{d}^{\left(q\right)}$$

where $E[v_{d}^{\left(q\right)}]$ and $\mathrm{Var}[v_{d}^{\left(q\right)}]$ are the mean and variance of the *d*-th channel computed over the mini-batch, $\varepsilon =10^{-5}$ is a small constant for numerical stability, and $\lambda_{d}^{\left(q\right)}$ and $\psi_{d}^{\left(q\right)}$ are learnable scale and shift parameters, respectively.

**ReLU Activation:** The ReLU activation function introduces nonlinearity:

(14) $$y_{d,t}^{\left(q\right)}=\max\left(0,{\tilde{v}}_{d,t}^{\left(q\right)}\right)$$

**Average Pooling Layer:** For the first and second convolutional modules (q=1,2), an average pooling layer is applied to perform local smoothing:

(15) $$u_{d,t}^{\left(q\right)}=\frac{1}{\kappa_{pool}}\sum_{f=0}^{\kappa -1}y_{d,t+f}^{\left(q\right)}$$

where $\kappa_{pool}$ denotes the pooling window size and *f* is the position index within the pooling window. In this work, $\kappa_{pool}=2$ with stride 1 and same padding, so the temporal dimension remains unchanged. For the third module (q=3), no pooling is applied, and the output after ReLU is directly denoted as $y^{\left(3\right)}$.

The output $y^{\left(3\right)}$ of the third convolutional module has dimensions 512×2β, which is subsequently fed into the global average pooling module.

#### 3.2.2. Global Average Pooling Module

Following the third convolutional module, the feature maps $y^{\left(3\right)}$ are processed by the global average pooling (GAP) module, which comprises a flatten layer, a GAP layer, and a dropout layer.

The flatten layer reshapes the feature tensor by collapsing spatial dimensions. The subsequent GAP layer then aggregates features across the temporal dimension. For each feature channel, the GAP operation is expressed as

(16) $$\zeta_{c^{\prime }}=\frac{1}{T}\sum_{t^{\prime }=1}^{T}y_{c^{\prime }}\left(t^{\prime }\right)$$

where $y_{c^{\prime }}\left(t^{\prime }\right)$ represents the feature value of the $c^{\prime }$-th channel at position $t^{\prime }$, *T* is the feature length, and $\zeta_{c^{\prime }}$ is the aggregated output for channel $c^{\prime }$.

**Figure 5:**
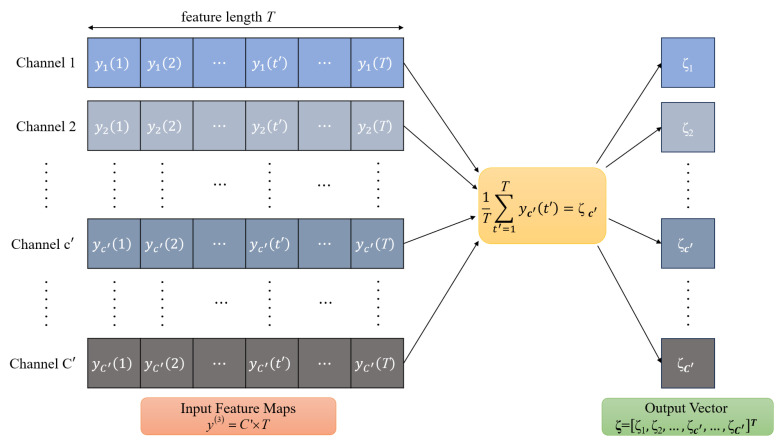
Working process of GAP layer in DNN classifier.

Figure 5 illustrates the GAP operation process. As shown in the figure, the input feature maps $y^{\left(3\right)}$ consist of $C^{\prime }$ channels (with $C^{\prime }=512$), and each channel has a feature length *T*, which is formulated as T=2β. For each channel $c^{\prime }$, the GAP layer computes the arithmetic mean of all feature values $y_{c^{\prime }}(1),y_{c^{\prime }}(2),\ldots ,y_{c^{\prime }}(T)$ along the feature length dimension, producing a single scalar output $\zeta_{c^{\prime }}$. This operation is performed independently for all $C^{\prime }$ channels, transforming the input tensor of size $C^{\prime }\times T$ into a compact output vector of length $C^{\prime }$. The combination of flattening and global average pooling transforms the hierarchical feature maps into a compact fixed-length feature vector, significantly reducing the number of parameters passed to the fully connected layers while preserving the most representative information from each channel.

A dropout layer with drop rate $\delta^{\left(0\right)}=0.3$ is then applied for regularization:

(17) $$\rho^{\left(0\right)}=\mathrm{Dropout}\left(\zeta ,\delta^{\left(0\right)}\right)$$

where $\rho^{\left(0\right)}$ is the output of the GAP module, which serves as the input to the subsequent fully connected modules.

#### 3.2.3. Fully Connected Modules

The feature vector $\rho^{\left(0\right)}$ is subsequently passed through two FC modules for high-level feature transformation. Each FC module comprises a FC layer, a BN layer, a ReLU activation function, and a dropout layer. As the BN, ReLU, and dropout operations have been described in Section 3.2.1 and Section 3.2.2, only the FC layer operation is elaborated upon here.

For the *h*-th FC module (h=1,2), the FC layer performs a linear transformation:

(18) $$o^{\left(h\right)}=M^{\left(h\right)}\rho^{\left(h-1\right)}+\omega^{\left(h\right)}$$

where $M^{\left(h\right)}$ is the weight matrix, $\omega^{\left(h\right)}$ is the bias vector, and $\rho^{\left(h-1\right)}$ is the input to the *h*-th module. The output $o^{\left(h\right)}$ of the *h*-th FC layer then passes through the BN, ReLU, and dropout layers sequentially, producing $\rho^{\left(h\right)}$.

#### 3.2.4. Classification Module

The classification module receives the output $\rho^{\left(2\right)}$ from the second FC module and produces the final joint prediction of channel quality and transmitted bit. It comprises a FC layer, a softmax layer, and an output classification layer.

The fully connected layer maps the 256-dimensional feature vector to four output neurons corresponding to the four classes:

(19) $$g=M^{\left(3\right)}\rho^{\left(2\right)}+\omega^{\left(3\right)}$$

where $g={\left[g_{0},g_{1},g_{2},g_{3}\right]}^{T}$ is the output vector.

The softmax layer converts the output into a probability distribution:

(20) $$p_{I=i}=\frac{e^{g_{i}}}{\sum_{k=0}^{3}e^{g_{k}}},\;i\in \left\{0,1,2,3\right\}$$

where $p_{I=i}$ represents the probability that the input signal belongs to class *i*.

The classification layer determines the predicted label:

(21) $$I=\arg\max_{i\in \{0,1,2,3\}}p_{I=i}$$

As described in Section 2.1, the demodulated bit can be recovered by ${\hat{b}}_{m}=I\;\mathrm{mod}\;2$.

### 3.3. The Parameter Configuration and Training Process of DNN Classifier

#### 3.3.1. Dataset for Training

In wireless communications, a multipath Rayleigh fading channel is a stochastic process, and each randomly generated set of channel parameters—including the path fading coefficients and delays—is referred to as a *channel realization*, representing the instantaneous channel state at a given moment. Different channel realizations exhibit distinct fading characteristics, giving rise to varying transmission quality.

The training dataset is constructed as follows. A total of Ξ independent channel realizations are generated. For the ξ-th realization (ξ=1,2,…,Ξ), a multipath Rayleigh fading channel $\Gamma_{\xi }$ and an $E_{b}/N_{0}$ value randomly selected within a predefined range are generated. Under the quasi-static fading assumption, the channel coefficients $\Gamma_{\xi }$ remain constant throughout the entire evaluation period for each realization.

To evaluate the transmission quality of each channel realization, $N_{s}$ DCSK-modulated symbols are consecutively transmitted through the fixed channel $\Gamma_{\xi }$, where each symbol experiences identical channel fading but is corrupted by independent AWGN samples. After demodulation, the BER of the ξ-th channel realization is computed as

(22) $$BER_{\xi }=\frac{1}{N_{s}}\sum_{m^{\prime }=1}^{N_{s}}\left|b_{m^{\prime }}-{\hat{b}}_{m^{\prime }}\right|$$

where $b_{m^{\prime }}$ and ${\hat{b}}_{m^{\prime }}$ represent the transmitted and demodulated bits of the $m^{\prime }$-th symbol, respectively, and $N_{s}$ is the number of symbols used to evaluate each channel realization.

Based on the BER values of all Ξ channel realizations, each channel is classified as either high quality or low quality. To determine the optimal classification criterion, a systematic parametric study is conducted by varying the classification threshold ratio Δ. Specifically, Δ defines the percentile boundary between the two classes: channel realizations whose BER exceeds the Δ-th percentile are labeled as low-quality, while the remaining (1−Δ) fraction with lower BER values are labeled as high-quality.

The threshold Δ is evaluated across the following candidate values for each spreading factor:

- **For** β=40: Δ∈{40%,50%,60%}
- **For** β=80: Δ∈{20%,30%,40%}

**Figure 6:**
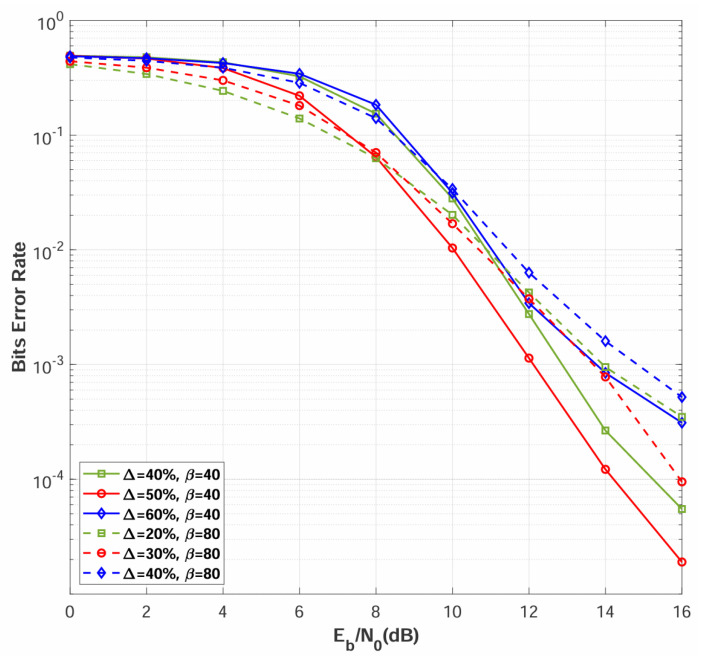
BER performance comparisons of different classification threshold ratios Δ over multipath Rayleigh fading channel, with N=2.

For each channel realization ξ, the received signal $r_{\xi }$ is stored as the DNN input, and its label $I_{\xi }$ is determined by the combination of channel quality and transmitted bit, as specified in Table 1. All simulations are conducted with N=2 relay nodes over multipath Rayleigh fading channels, and the results are presented in Figure 6. The results indicate that Δ=50% yields the best BER performance for β=40, while Δ=30% is optimal for β=80.

The classification threshold Δ influences demodulation performance through two primary mechanisms: (1) **Training data balance**—excessively small or large Δ values introduce class imbalance, causing the network to develop prediction bias toward the majority class and degrading generalization performance; (2) **Inter-class separability**—an optimal Δ maximizes the BER gap between the two classes, enabling the network to learn robust decision boundaries, whereas an inappropriate Δ reduces class separability and increases misclassification rates. The identified optimal thresholds (Δ=50% for β=40 and Δ=30% for β=80) represent the operating points that minimize classification errors while preserving adequate sample diversity across both classes.

Through this design, the network not only learns to distinguish binary symbol values but also implicitly estimates the instantaneous channel condition, achieving the joint detection of transmitted bits and channel quality within a unified DNN framework.

The dataset partitioning strategy and associated training overhead are summarized as follows. For each spreading factor β, the full dataset comprises 500,000 training samples, 100,000 validation samples, and 200,000 test samples, yielding a total of 800,000 samples with a split ratio of approximately 62.5%/12.5%/25%. The training set is used to optimize network parameters via backpropagation; the validation set supports early stopping and hyperparameter tuning to mitigate overfitting; and the test set is reserved for final performance evaluation under unseen channel realizations. All experiments are conducted in MATLAB R2024b on an NVIDIA RTX 4090Ti GPU with CUDA 12.1 acceleration. Under this configuration, the complete training process requires approximately 30 min, confirming that the offline training cost of the proposed DNN classifier is moderate and practically acceptable.

#### 3.3.2. Selection of Hyper-Parameters

Hyperparameter optimization plays a critical role in determining the accuracy, convergence speed, and computational complexity of the DNN model during training. Appropriate hyperparameter selection is therefore essential for maximizing the classification accuracy of the proposed DNN-based classifier.

*(i)* OutputDimensionandTrainableParameters: Table 2 summarizes the output dimensions and trainable parameter counts for each layer of the proposed DNN-based classifier. These parameters directly influence the model’s capacity and computational complexity. Two key parameters that significantly affect the network’s feature extraction capability are the kernel size $\gamma^{\left(q\right)}$ and the number of output channels $C_{out}^{\left(q\right)}$ (i.e., the number of filters) of the convolutional layers. In this work, these parameters are set as $\gamma^{\left(1\right)}=16$, $\gamma^{\left(2\right)}=8$, $\gamma^{\left(3\right)}=4$ and $C_{out}^{\left(1\right)}=128$, $C_{out}^{\left(2\right)}=256$, $C_{out}^{\left(3\right)}=512$. These values are determined through empirical experimentation with due consideration of available computational resources. The chosen configuration allows the convolutional layers to effectively capture the temporal patterns in the received chaotic signals without incurring excessive computational overhead.

*(ii)* LearningRate: The network is optimized using the Adam optimizer, which adaptively adjusts the learning rate during training, potentially leading to faster convergence compared to traditional stochastic gradient descent [37]. The initial learning rate is set to $10^{-3}$, providing a stable yet efficient convergence [38]. The model is trained for a maximum training cycle of 20 epochs, incorporating an early stopping algorithm to prevent overfitting and optimize computational resources. This dynamic adjustment prevents premature convergence and ensures the fine-tuning of network parameters in later training stages. The batch size is set to 128 to balance gradient estimation stability and computational efficiency. To avoid gradient explosion during backpropagation, we apply gradient clipping on the norm of gradients, setting the clipping threshold to 1.0—a widely used strategy to stabilize training in deep neural networks [39].

*(iii)* LossFunction: Since the demodulation task is formulated as a four-class classification problem, the categorical cross-entropy loss, which is particularly effective for multi-class classification tasks [40], is adopted as the optimization objective. For a given input signal $r_{\xi }$ with true label $I_{\xi }$, the loss function is expressed as

(23) $$L\left(\theta \right)=-\frac{1}{\Xi }\sum_{\xi =1}^{\Xi }\sum_{i=0}^{3}\eta_{\xi ,i}\log\left(p_{I=i}\right)$$

where θ denotes the set of all trainable parameters in the DNN, Ξ is the total number of training samples, $p_{I=i}$ is the predicted probability that the input belongs to class *i*, and $\eta_{\xi ,i}$ denotes the *i*-th element of the one-hot encoded label for the ξ-th sample, which equals 1 if $I_{\xi }=i$ and 0 otherwise. This loss function measures the divergence between the predicted probability distribution and the true one-hot encoded label, effectively guiding the network to minimize classification errors across all categories. The combination of Adam optimization and cross-entropy loss ensures efficient convergence and high reliability in the classification of chaotic signals under different channel conditions.

*(iv)* $E_{b}/N_{0}$: The selected signal-to-noise ratio (SNR) of energy per bit to noise power spectral density ($E_{b}/N_{0}$) at the training stage will generate the corresponding datasets, and affect the BER performance of MR-DL-DCSK communication system at the online deployment stage indirectly. In the training, we do not use a certain $E_{b}/N_{0}$ value to generate datasets, but choose a range of $E_{b}/N_{0}$ to generate datasets in a balanced manner for avoiding the problem of overfitting or underfitting [41]. In this article, the range of $E_{b}/N_{0}$ is [13,15] dB for training.

### 3.4. Complexity Analysis

This section presents a computational complexity analysis of the proposed DNN-based receiver in the multi-relay DL-DCSK system. To ensure fair evaluation, we adopt the same complexity analysis method as employed in [29,42]. Specifically, the computational complexity is defined as the total number of operations per symbol performed at the receiver, expressed in terms of the asymptotic upper bound O(·). Since the DNN training is conducted offline, the complexity analysis focuses exclusively on the online deployment stage, where the proposed intelligent classifier performs real-time signal processing.

As described in Section 2.1, each transmitted bit is represented by two chaotic sequences of length β, resulting in an input sequence length of 2β. Due to the complex input splitting, the actual input dimension becomes $N_{in}=4\beta$. Let $F_{\ell }$ and $S_{\ell }$ denote the number of filters and kernel size of the *ℓ*-th convolutional layer respectively, $N_{fc,\mu }$ represent the number of neurons in the μ-th fully connected layer, and $N_{bn}$ denote the number of batch normalization layers.

For the proposed DNN-based classifier, the computational complexity of each component can be expressed as follows. The three 1D convolutional layers contribute $O(\sum_{\ell =1}^{3}N_{in}\cdot F_{\ell }\cdot S_{\ell }\cdot F_{\ell -1})$, the batch normalization layers contribute $O(N_{in}\cdot \sum_{\ell =1}^{N_{bn}}F_{\ell })$, the pooling layers contribute $O(N_{in}\cdot \sum_{\ell =1}^{3}F_{\ell })$, and the fully connected layers contribute $O(\sum_{\mu =1}^{3}N_{fc,\mu -1}\cdot N_{fc,\mu })$. Therefore, the total complexity of the proposed system is given by

(24) $$O_{total}=O\left(N_{in}\cdot \Omega_{conv}+N_{in}\cdot \Omega_{bn}+N_{in}\cdot \Omega_{pool}+\Omega_{fc}\right),$$

where $\Omega_{conv}$, $\Omega_{bn}$, $\Omega_{pool}$, and $\Omega_{fc}$ represent the aggregated complexity coefficients for convolutional, batch normalization, pooling, and fully connected layers, which are defined as

(25) $$\Omega_{conv}=\sum_{\ell =1}^{3}F_{\ell }\cdot S_{\ell }\cdot F_{\ell -1},$$

(26) $$\Omega_{bn}=\sum_{\ell =1}^{N_{bn}}F_{\ell },$$

(27) $$\Omega_{pool}=\sum_{\ell =1}^{3}F_{\ell },$$

(28) $$\Omega_{fc}=\sum_{\mu =1}^{3}N_{fc,\mu -1}\cdot N_{fc,\mu }.$$

To provide a concrete example, we consider the configuration where β=40, corresponding to an input sequence length of $N_{in}=4\beta =160$. By substituting the network parameters, the computational operations per symbol for the proposed multi-relay DL-DCSK receiver can be approximated as $127.0\times 10^{6}$ operations. When β=80 (i.e., $N_{in}=320$), this increases to approximately $253.5\times 10^{6}$ operations.

Compared to traditional DCSK receivers, which typically exhibit a complexity of O(β) for correlation-based detection, our proposed DNN-based receiver demonstrates higher computational demands. However, this increased complexity is justified by the superior BER performance, as evidenced by our simulation results. Furthermore, the computational burden can be significantly mitigated through parallel processing on modern hardware architectures such as GPUs and dedicated AI accelerators, making the proposed scheme practical for real-time applications.

Beyond the theoretical complexity analysis, we further conduct practical detection latency measurements to provide a more intuitive evaluation of the computational overhead. All experiments are performed on MATLAB R2024b with an NVIDIA RTX 4090Ti GPU accelerated by CUDA 12.1. For each configuration, the per-symbol detection latency is averaged over 10,000 independent trials to ensure statistical reliability. The measured detection latencies of the proposed DNN-based receiver and the conventional EGC-DCSK receiver are summarized in Table 3.

As shown in Table 3, the conventional EGC-DCSK receiver exhibits detection latency on the order of $10^{-2}$ ms, with the latency for β=80 slightly exceeding that for β=40, which is consistent with the linear computational complexity O(β) of correlation-based detection. In contrast, the DNN-based receiver incurs a per-symbol latency of approximately 7.5–7.9 ms. Notably, the DNN detection latency remains approximately constant across different spreading factors, indicating that the total inference time is dominated by the fixed GPU scheduling overhead and the fully connected layers with constant parameter counts, rather than the convolutional operations whose computational load scales with β.

Although the DNN-based receiver introduces substantially higher detection latency compared to the conventional receiver, several important considerations should be noted. First, the measured latency reflects the unoptimized MATLAB deep learning inference environment. In practical deployment scenarios, employing dedicated inference engines such as TensorRT or ONNX Runtime with model optimization techniques including operator fusion, mixed-precision (FP16/INT8) quantization, and batch inference can reduce the inference latency by one to two orders of magnitude [43]. Second, regarding power consumption, the NVIDIA RTX 4090Ti GPU used in this work has a rated thermal design power (TDP) of 450 W, which is significantly higher than that of conventional low-power processors used for traditional DCSK receivers. However, the increasing maturity and availability of edge AI accelerators, such as the NVIDIA Jetson Orin series with a TDP of merely 15–60 W, provide a viable pathway for deploying the proposed DNN-based receiver on resource-constrained and energy-sensitive platforms, such as IoT devices and vehicular communication terminals. Third, model compression techniques including network pruning, knowledge distillation, and weight quantization can further reduce both the computational latency and power consumption without significant degradation in detection accuracy [44]. Therefore, the proposed DNN-based receiver, while incurring higher computational cost than conventional approaches, offers a favorable trade-off between detection performance and computational overhead, and its practical deployment is well supported by modern AI hardware and software ecosystems.

## 4. Simulation Results and Discussions

In this section, results on BER are presented to demonstrate the superiority of the proposed multi-relay DL-DCSK system over multipath Rayleigh fading channels. Without loss of generality, the maximum number of relays is set to N=4 in simulations. Unless otherwise specified, in the simulations it is assumed that all the wireless channels have three paths (i.e., $L_{SR_{n}}=L_{R_{n}D}=3$) and the power-delay profile is set as follows:

1. For the $S\to R_{1}$ link, $E[\alpha_{SR_{1},1}^{2}]=1/3$, $E[\alpha_{SR_{1},2}^{2}]=1/3$, $E[\alpha_{SR_{1},3}^{2}]=1/3$, $\tau_{SR_{1},1}=0$, $\tau_{SR_{1},2}=2$, $\tau_{SR_{1},3}=4$;
2. For the $S\to R_{2}$ link, $E[\alpha_{SR_{2},1}^{2}]=1/5$, $E[\alpha_{SR_{2},2}^{2}]=1/2$, $E[\alpha_{SR_{2},3}^{2}]=3/10$, $\tau_{SR_{2},1}=0$, $\tau_{SR_{2},2}=3$, $\tau_{SR_{2},3}=6$;
3. For the $S\to R_{3}$ link, $E[\alpha_{SR_{3},1}^{2}]=1/2$, $E[\alpha_{SR_{3},2}^{2}]=1/3$, $E[\alpha_{SR_{3},3}^{2}]=1/6$, $\tau_{SR_{3},1}=0$, $\tau_{SR_{3},2}=3$, $\tau_{SR_{3},3}=5$;
4. For the $S\to R_{4}$ link, $E[\alpha_{SR_{4},1}^{2}]=1/4$, $E[\alpha_{SR_{4},2}^{2}]=2/5$, $E[\alpha_{SR_{4},3}^{2}]=7/20$, $\tau_{SR_{4},1}=0$, $\tau_{SR_{4},2}=2$, $\tau_{SR_{4},3}=6$;
5. For the $R_{1}\to D$ link, $E[\alpha_{R_{1}D,1}^{2}]=1/4$, $E[\alpha_{R_{1}D,2}^{2}]=1/4$, $E[\alpha_{R_{1}D,3}^{2}]=1/2$, $\tau_{R_{1}D,1}=0$, $\tau_{R_{1}D,2}=4$, $\tau_{R_{1}D,3}=8$;
6. For the $R_{2}\to D$ link, $E[\alpha_{R_{2}D,1}^{2}]=1/3$, $E[\alpha_{R_{2}D,2}^{2}]=2/5$, $E[\alpha_{R_{2}D,3}^{2}]=4/15$, $\tau_{R_{2}D,1}=0$, $\tau_{R_{2}D,2}=5$, $\tau_{R_{2}D,3}=10$;
7. For the $R_{3}\to D$ link, $E[\alpha_{R_{3}D,1}^{2}]=1/5$, $E[\alpha_{R_{3}D,2}^{2}]=2/15$, $E[\alpha_{R_{3}D,3}^{2}]=2/3$, $\tau_{R_{3}D,1}=0$, $\tau_{R_{3}D,2}=1$, $\tau_{R_{3}D,3}=3$;
8. For the $R_{4}\to D$ link, $E[\alpha_{R_{4}D,1}^{2}]=3/5$, $E[\alpha_{R_{4}D,2}^{2}]=1/15$, $E[\alpha_{R_{4}D,3}^{2}]=1/3$, $\tau_{R_{4}D,1}=0$, $\tau_{R_{4}D,2}=3$, $\tau_{R_{4}D,3}=7$.

Moreover, assume that the distance of the $S\to R_{n}$ and $R_{n}\to D$ ( n∈{0,1,2,3} ) link is constant, and the path loss is ignored.

To evaluate the effectiveness of the proposed multi-relay DL-DCSK scheme, comprehensive BER performance comparisons are conducted against two benchmark schemes. The first is the Two-Way Relay Network-Coded DCSK (TWRNC-DCSK) system [16]. The second benchmark is a fully connected DNN (FC-DNN) architecture proposed in [45] for received symbol detection. This FC-DNN-based detection method serves as a representative data-driven baseline, against which the performance advantage of the proposed multi-relay DL-DCSK system in terms of detection accuracy is assessed.

### 4.1. BER Comparison Among Different Spreading Factor β over Rayleigh Channel

**Figure 7:**
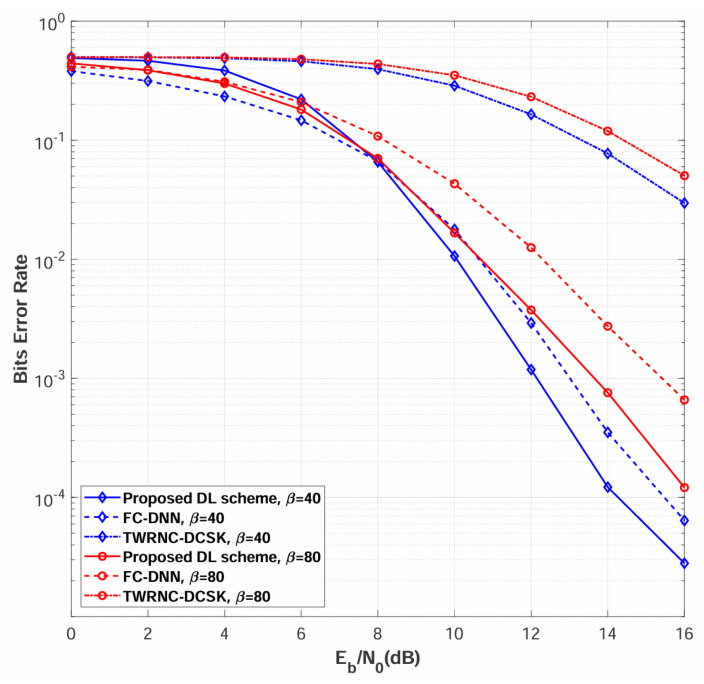
BER performance comparisons between multi-relay DL-DCSK, TWRNC-DCSK, and FC-DNN-based detection method over multipath Rayleigh fading channels. The spreading factors are β = 40 and 80.

Figure 7 compares the BER performance of the proposed multi-relay DL-DCSK system against the benchmark schemes over multipath Rayleigh fading channels, with the number of relays set to N=2 and spreading factors of β=40 and β=80. The simulation results demonstrate that the proposed multi-relay DL-DCSK scheme achieves substantial performance improvements over both traditional TWRNC-DCSK and FC-DNN detection methods across all evaluated spreading factors and $E_{b}/N_{0}$ conditions. Furthermore, BER performance is observed to degrade with increasing β, a trend consistent with those reported in both conventional DCSK systems and DL-aided chaotic communication schemes [26,27,28,29,30].

The proposed multi-relay DL-DCSK scheme outperforms the FC-DNN-based detection method due to fundamental architectural differences in signal processing. Specifically, the convolutional layers preserve the temporal correlation structure of chaotic sequences, extract spatially invariant features through local receptive fields, and capture hierarchical multi-scale patterns, whereas FC-DNN flattens the input signal into a vector representation, destroying the temporal structure, treating dimensions independently, and losing the sequential dependencies essential for chaotic signal detection. The DL-based detection schemes substantially outperform TWRNC-DCSK scheme due to advanced receiver architecture. The DL-based receivers learn optimal nonlinear decision boundaries through deep neural networks that extract robust features from multipath-distorted chaotic signals, perform implicit channel equalization, and adapt to the statistics inherent in DCSK correlation-based demodulation, whereas TWRNC-DCSK scheme employs conventional correlation receivers that use linear correlation followed by hard thresholding—a processing approach that is fundamentally suboptimal for chaotic signals in multipath fading channels, lacks equalization mechanisms, and generates relay decoding errors that cascade through the decode-and-forward protocol to degrade the overall system performance.

### 4.2. BER Comparison Among Different Numbers of Relays over Rayleigh Channel

**Figure 8:**
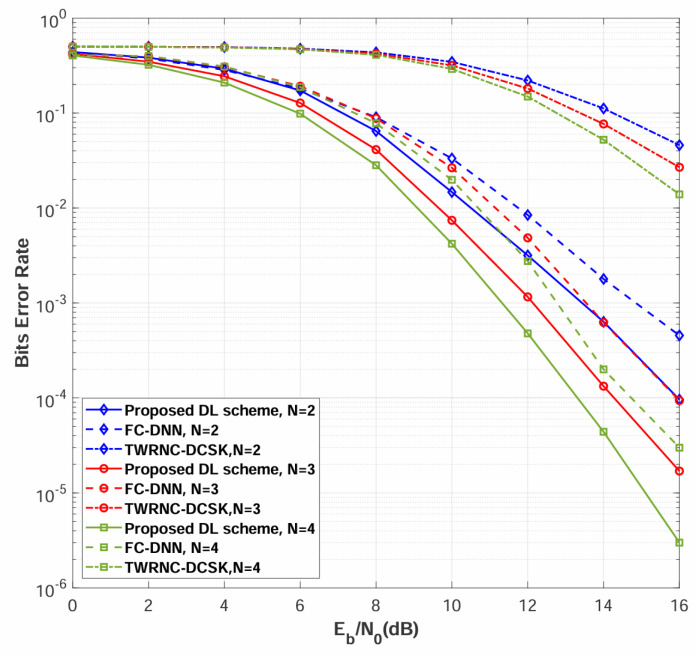
BER performance comparisons between multi-relay DL-DCSK, TWRNC-DCSK, and FC-DNN-based detection method over multipath Rayleigh fading channels, where the power-delay profiles for different sub-channels are identical, and the spreading factor is β = 80.

Simulations are conducted to evaluate the BER performance of the multi-relay DL-DCSK system among different numbers of relays. Figure 8 compares the BER performance of the proposed multi-relay DL-DCSK scheme and the benchmark systems over multipath Rayleigh fading channels with different numbers of relays, where the channel parameters are set to $E\left[\alpha_{SR_{n},1}^{2}\right]=E\left[\alpha_{SR_{n},2}^{2}\right]=E\left[\alpha_{SR_{n},3}^{2}\right]=E\left[\alpha_{R_{n}D,1}^{2}\right]=E\left[\alpha_{R_{n}D,2}^{2}\right]=E\left[\alpha_{R_{n}D,3}^{2}\right]=1/3$, $\tau_{SR_{n},1}=\tau_{R_{n}D,1}=0$, $\tau_{SR_{n},2}=\tau_{R_{n}D,2}=2$, $\tau_{SR_{n},3}=\tau_{R_{n}D,3}=5$ for all links. As shown in Figure 8, the proposed multi-relay DL-DCSK scheme consistently outperforms conventional TWRNC-DCSK scheme and FC-DNN detection methods across all evaluated scenarios.

**Figure 9:**
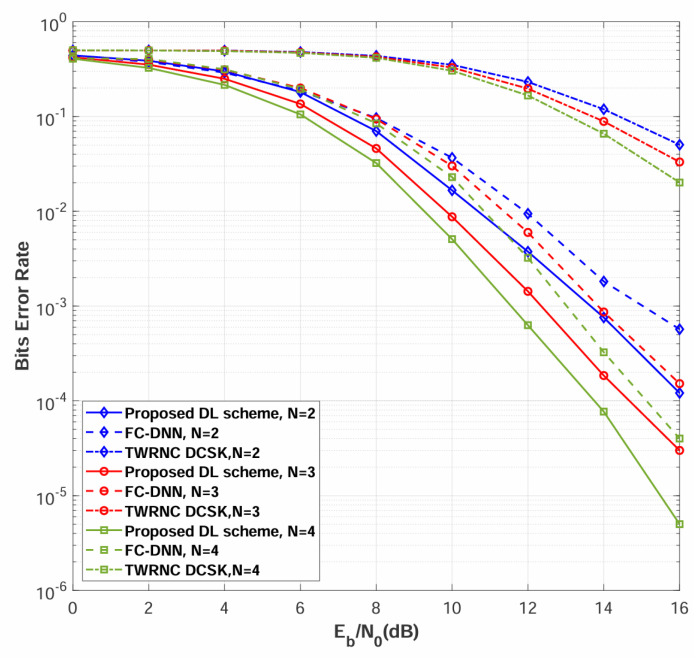
BER performance comparisons between multi-relay DL-DCSK, TWRNC-DCSK, and FC-DNN-based detection method over multipath Rayleigh fading channels, where the power-delay profiles for different sub-channels are different, and the spreading factor is β = 80.

Figure 9 presents the BER performance comparison when the power-delay profiles for different sub-channels are different. The detailed power-delay profiles in this figure are the same as those listed in the first paragraph of Section 4. As can be observed, the relative performance among different values of *N* in this figure remains the same as that in Figure 8. The results demonstrate that the proposed multi-relay DL-DCSK scheme maintains its performance advantage over both the TWRNC-DCSK and FC-DNN-based detection methods.

Both Figure 8 and Figure 9 demonstrate consistent performance improvement as the number of relay nodes increases from N=2 to N=4. This phenomenon stems from cooperative spatial diversity [46,47], where multiple independent relay paths provide redundant signal replicas that collectively enhance detection reliability. The minimal performance difference between identical and different channel configurations demonstrates the robustness of the DL-based approach to channel variability, confirming its suitability for realistic wireless environments.

### 4.3. Performance Comparison Among Different Relay Coordination Strategies

To validate the effectiveness of the proposed channel quality-aware relay coordination strategy introduced in Section 2.2.1, a comprehensive ablation study is conducted under controlled and identical system configurations. All compared schemes share the same multi-relay DL-DCSK system topology illustrated in Figure 2, with N=2 relay nodes and a spreading factor of β=80. The channel parameters follow the power-delay profiles specified in Section 4, where distinct profiles are assigned to different sub-channels.

Four relay coordination strategies are evaluated and compared:

**Strategy 1 (EGC, DCSK):** This baseline scheme employs the conventional DCSK transceiver structure at both the transmitter and receiver ends. All relays independently decode and forward their demodulated bits using the DF protocol without any bit alignment. At the destination, equal gain combining (EGC) is applied to merge the received signals from all relays before final demodulation.

**Strategy 2 (BER-based, DCSK):** This scheme maintains the conventional DCSK transceiver structure while implementing the BER-based relay selection strategy proposed in [4]. Since this method requires CSI to calculate the instantaneous signal-to-noise ratio (SNR) at the receiver, we calculate the SNR using the method from [48], which requires CSI, and thus we assume perfect CSI is available. Only the selected relay transmits in this scheme, thus no diversity combining is required at the destination.

**Strategy 3 (Proposed DL scheme based BER):** This configuration adopts the proposed DNN-based receiver structure described in Section 2-A for signal demodulation at both relays and destination. However, the relay nodes do not utilize the DNN’s probability distribution output $p_{I}=\left[p_{I=0},p_{I=1},p_{I=2},p_{I=3}\right]$ for decoded bit alignment. Instead, this scheme employs the same BER-based relay selection strategy as Strategy 2 for forwarding decisions, with relays independently forwarding their demodulated bits.

**Strategy 4 (Proposed DL scheme):** This represents our complete proposed system as detailed in Section 2.2.1.

**Figure 10:**
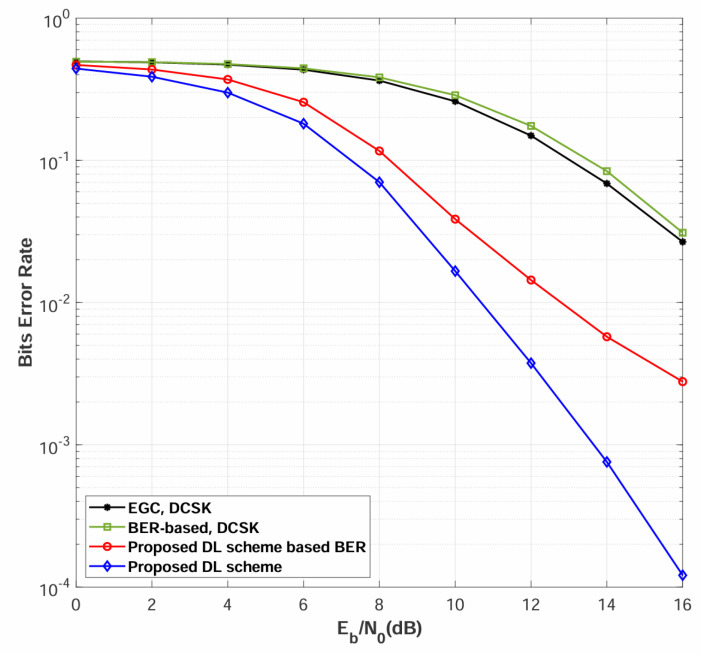
BER performance comparisons among different relay coordination strategies over multipath Rayleigh fading channels, where the number of relays is N=2, the power-delay profiles for different sub-channels are different, and the spreading factor is β=80.

Figure 10 presents the BER performance comparison between the proposed strategy and the other three strategies over multipath Rayleigh fading channels. Several important observations can be drawn from the results:

The proposed scheme (Strategy 4) achieves the best performance among all evaluated strategies across the entire $E_{b}/N_{0}$ range. Compared to Strategy 3, the proposed channel quality-based relay coordination provides an additional 3.5 dB performance gain at BER = $3\times 10^{-3}$. This significant improvement validates our key innovation: by leveraging the DNN channel quality probability distribution rather than relying on explicit CSI-based SNR estimation, the proposed relay alignment and selection mechanism can more accurately identify and utilize the most reliable transmission paths. The relay bit alignment process ensures that all relays forward consistent information derived from the highest-quality received signal, thereby maximizing spatial diversity gains while minimizing error propagation.

Furthermore, Strategy 3 exhibits substantially better BER performance compared to Strategy 2, with approximately 5.5 dB gain at BER = $3\times 10^{-2}$. This demonstrates the effectiveness of the DNN-based receiver in extracting robust features from chaotic signals.

The performance gap between the proposed scheme and the other strategies widens as $E_{b}/N_{0}$ increases, demonstrating that the proposed approach is particularly advantageous in scenarios where high reliability is critical. The results validate that the joint design of DNN-based channel quality assessment and probability-guided relay coordination constitutes an effective approach for multi-relay cooperative chaotic communications.

### 4.4. Generalization Performance

#### 4.4.1. BER Performance over Quasi-Static V2V Channels

To evaluate the generalization capability of the proposed multi-relay DL-DCSK system, simulations are conducted over channel conditions distinct from those encountered during offline training. Specifically, the DNN classifier is trained exclusively on multipath Rayleigh fading channels, while the testing is performed over vehicle-to-vehicle (V2V) communication channels modeled by the double-Gamma (dGG) distribution. This cross-environment evaluation assesses the robustness and adaptability of the proposed system when deployed under previously unseen propagation conditions.

The dGG distribution is suitable for modeling non-homogeneous double-scattering radio propagation fading conditions, which can be frequently observed in V2V communications [49]. Unlike the Rayleigh fading model employed during training, the dGG channel exhibits non-Rayleigh amplitude statistics and distinct fading characteristics that more faithfully reflect the complexities of real-world vehicular propagation environments.

**Figure 11:**
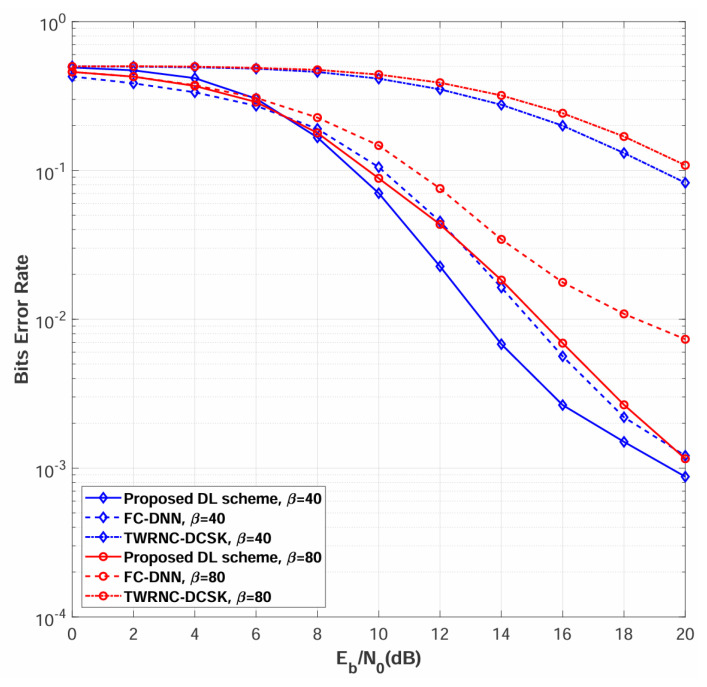
BER performance comparisons between the multi-relay DL-DCSK, TWRNC-DCSK, and FC-DNN-based detection methods over V2V communication channels. The spreading factors are β = 40 and 80.

Figure 11 compares the BER performance of the proposed multi-relay DL-DCSK scheme against benchmark systems over V2V communication channels, with N=2 relay nodes and spreading factors of β=40 and β=80. Despite being trained exclusively on Rayleigh fading channels, the proposed multi-relay DL-DCSK scheme demonstrates superior BER performance compared to the TWRNC-DCSK system and the FC-DNN-based detection method. This result validates the strong generalization capability of the proposed DNN architecture.

**Figure 12:**
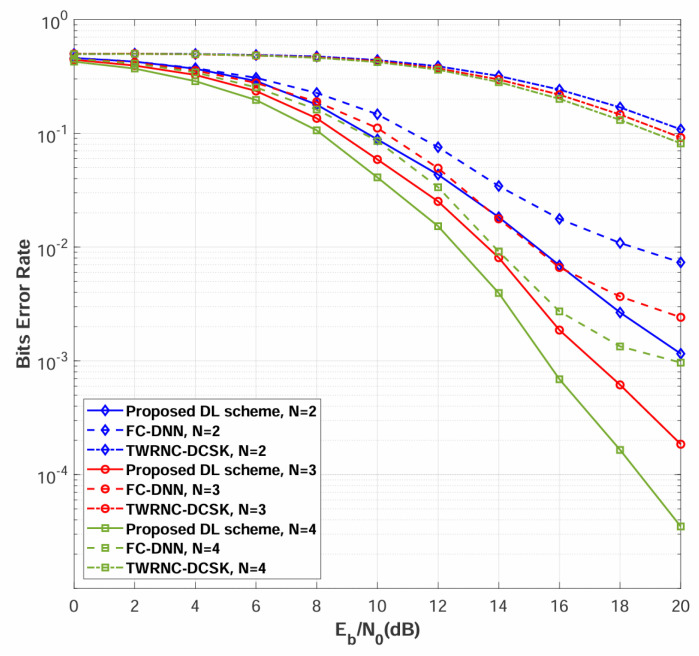
BER performance comparisons between multi-relay DL-DCSK, TWRNC-DCSK, and FC-DNN-based detection method over V2V communication channels, where the spreading factor is β = 80.

Figure 12 further investigates the generalization performance with different numbers of relays over V2V communication channels, where the spreading factor is set to β=80. Similar trends to those observed under Rayleigh fading conditions are evident, where increasing the relay count *N* leads to progressive performance improvements. The proposed scheme maintains its superiority over the TWRNC-DCSK system and the FC-DNN-based detection method throughout all configurations.

These results demonstrate that the proposed multi-relay DL-DCSK system exhibits excellent generalization capability. Although the DNN classifier is trained only on multipath Rayleigh fading channel data, it successfully learns robust feature representations that transfer effectively to the more complex V2V channel environment.

The strong generalization performance validates the practical applicability of the proposed scheme in real-world wireless communication scenarios, where channel conditions may vary significantly from the training environment. This is particularly important for applications such as intelligent transportation systems and IoT networks, where the receiver must operate reliably across diverse and time-varying propagation conditions.

#### 4.4.2. Impact of Doppler Shifts on BER Performance

To address the practical challenge of high mobility in V2V communication scenarios, the BER performance of the proposed multi-relay DL-DCSK system is further evaluated under high Doppler shift conditions. Specifically, simulations are conducted over V2V channels modeled by the double-generalized gamma (dGG) distribution with a relative vehicle speed of 80 km/h, which corresponds to a typical high-mobility highway scenario. The spreading factor is set to β=80, and the number of relays is varied as N∈{2,3,4}. It is important to note that the DNN classifier used in these simulations is trained exclusively on multipath Rayleigh fading channels without any Doppler-related features, making this a stringent test of the model’s generalization capability under channel dynamics that were entirely absent during training.

**Figure 13:**
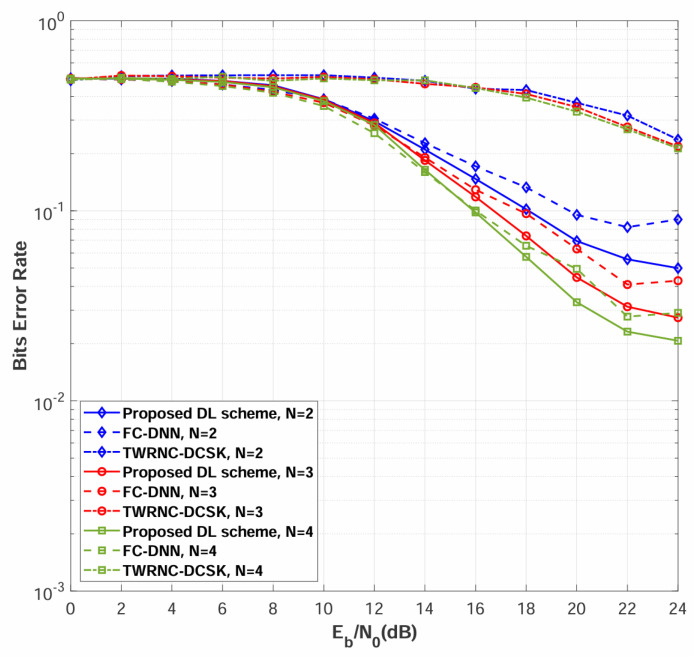
BER performance comparisons between multi-relay DL-DCSK, TWRNC-DCSK, and FC-DNN-based detection method over V2V communication channels with a relative vehicle speed of 80 km/h, where the spreading factor is β=80.

Figure 13 presents the BER performance comparison among the proposed multi-relay DL-DCSK scheme, the FC-DNN-based detection method, and the TWRNC-DCSK system under the 80 km/h V2V channel condition. Several important observations can be drawn from the results.

First, the conventional TWRNC-DCSK system exhibits a severe BER floor under high Doppler conditions. Across all relay configurations (N=2,3,4), the BER of TWRNC-DCSK remains above 0.3 even at $E_{b}/N_{0}=24$ dB, indicating that the conventional correlation-based receiver is essentially non-functional in this scenario. This degradation is attributed to the Doppler-induced time variation of the channel within each symbol period, which destroys the correlation between the reference and data-bearing chaotic sequences—the fundamental mechanism upon which conventional DCSK demodulation relies.

Second, the proposed multi-relay DL-DCSK scheme demonstrates significantly superior robustness against Doppler-induced channel variations. The BER curves of the proposed scheme maintain a consistent downward trend across the entire $E_{b}/N_{0}$ range. At $E_{b}/N_{0}=24$ dB, the proposed scheme achieves a BER of approximately $2\times 10^{-2}$ with N=4, $2.5\times 10^{-2}$ with N=3, and $6\times 10^{-2}$ with N=2. This robustness can be attributed to the fact that the DNN-based receiver learns implicit nonlinear feature representations from the received signal rather than relying on fixed correlation operations, making it inherently more resilient to Doppler-induced signal distortions.

Third, the FC-DNN-based detection method, while outperforming the TWRNC-DCSK system, exhibits inferior performance compared to the proposed scheme, particularly for N=2 where the BER curve tends to flatten at approximately $8\times 10^{-2}$ in the high $E_{b}/N_{0}$ regime. This performance saturation is likely caused by the inability of FC-DNN to capture the temporal correlation structure of Doppler-distorted chaotic sequences due to its fully connected architecture, which treats all input dimensions independently.

Fourth, increasing the number of relay nodes continues to yield progressive performance improvements under high Doppler conditions, consistent with the trends observed in the quasi-static channel scenarios presented in Section 4.2 and Section 4.4.1. This confirms that the proposed channel quality-aware relay coordination strategy remains effective in time-varying channel environments, successfully exploiting cooperative spatial diversity to enhance transmission reliability.

It is acknowledged that the absolute BER performance under 80 km/h conditions is degraded compared to the quasi-static and low-mobility scenarios, which is an expected consequence of the increased channel time variation. Nevertheless, the proposed scheme maintains a substantial performance advantage over both benchmark systems, demonstrating its practical viability for V2V communication applications. Furthermore, the current DNN model is trained exclusively on quasi-static Rayleigh fading channels without incorporating any Doppler-related features. Incorporating time-varying channel characteristics, such as Doppler-shifted signal samples, into the training dataset represents a promising direction for further improving the system’s performance under high-mobility conditions. Moreover, as observed in the above results, the fixed DNN architecture exhibits varying degrees of performance degradation across different Doppler regimes, suggesting that a single classifier may face difficulties in simultaneously handling diverse channel dynamics. In this regard, the Mixture-of-Experts (MoE)-based reinforcement learning framework proposed in [50], which employs specialized expert networks to handle distinct regions of the state space and dynamically aggregates their outputs via a gating network, offers a promising architectural direction for enabling adaptive and Doppler-regime-aware signal detection in future work.

## 5. Conclusions

This paper has presented a novel multi-relay DL-DCSK communication system that integrates a DNN-based intelligent classifier for joint channel quality assessment and symbol demodulation without requiring channel state information. Comprehensive simulation results over multipath Rayleigh fading channels demonstrate that the proposed multi-relay DL-DCSK system consistently outperforms the traditional TWRNC-DCSK system and the FC-DNN-based detection method. The system exhibits robust BER performance across varying spreading factors, relay counts, and power-delay profiles, with performance improving monotonically as the number of relays increases. Ablation experiments across different relay coordination strategies further validate the effectiveness of the proposed channel quality-aware relay coordination mechanism. Furthermore, the strong generalization capability demonstrated over V2V channels modeled by the double-Gamma (dGG) distribution confirms the potential applicability of the proposed system in propagation environments unseen during training. Simulations under high-mobility V2V conditions at a relative vehicle speed of 80 km/h confirm that the proposed system maintains robust performance against Doppler-induced channel variations. Practical detection latency measurements show that the DNN-based receiver incurs a per-symbol latency of approximately 7.5–7.9 ms in the unoptimized MATLAB environment, which can be substantially reduced through dedicated inference engines and model compression techniques for real-time deployment. Future work may explore extending the system to *M*-ary modulation schemes for higher spectral efficiency, incorporating Doppler-aware training strategies to enhance robustness under high-mobility conditions, and evaluating the proposed framework in broader practical mobile communication scenarios.

## Figures and Tables

**Table 1:** Correspondence between channel type, transmitted bit and label.

Channel Type	Transmitted Bit $b_{m}$	Corresponding Label *I*
High quality	“0”	“0”
High quality	“1”	“1”
Low quality	“0”	“2”
Low quality	“1”	“3”

**Table 2:** Output dimensions and trainable parameters of each module in the proposed DNN.

Layers	Output Dimension		Trainable Parameters
	β=40	β=80	
Input	2×80	2×160	0
1D-CL + BN + ReLU + AvgPool	128×80	128×160	4480
1D-CL + BN + ReLU + AvgPool	256×80	256×160	262,912
1D-CL + BN + ReLU	512×80	512×160	525,824
Flatten + GAP + Dropout	512	512	0
FC + BN + ReLU + Dropout	512	512	263,680
FC + BN + ReLU + Dropout	256	256	131,840
FC + Softmax	4	4	1028

**Table 3:** Detection latency comparison between DNN-based receiver and conventional EGC-DCSK receiver.

Receiver Type	Spreading Factor β	Latency (ms/Symbol)	Latency Ratio
DNN-based	40	7.8897	764.89×
EGC-DCSK	40	0.0103	
DNN-based	80	7.5620	647.68×
EGC-DCSK	80	0.0117	

## Data Availability

The original contributions presented in this study are included in the article. Further inquiries can be directed to the corresponding author.
